# Facial Neuralgia

**Published:** 1887-10

**Authors:** Almon Dewhirst


### Facial Neuralgia.

BY ALMON DEWHIRST, D.D.S.

The fact is well known that irritation of a nerve commonly manifests itself by pain at some point distant from, instead of at the seat of injury or disease. This phenomenon cannot be accounted for; it may be exemplified by the case of an inflamed liver giving rise to pain about the shoulder; a diseased hip joint producing pain at the knee, and an irritated tooth-pulp exciting pain at remote parts of the head and face.

Pain of this reflected or sympathetic character is called neuralgia. Neuralgia, or nerve pain, in a part may also occur without a lesion of any kind. It may be caused by debility, by cold, or by malaria, or it may accompany pregnancy. It may be due to disease of the great nerve centres, the brain, or spinal cord. Finally lesions of nerves not necessarily painful may give rise to neuralgia in consequence of disorder of the general health. Cases are met with frequently in which the diseased nerves of carious teeth, previously the seat of little or no pain, give rise to severe neuralgia when the vitality of the patient has become lowered by disease or by exhaustion.

Neuralgic pain is usually of a plunging, lancinating, or burning character, following the course of the nerve branches. It occurs in paroxysms, which are often regularly periodic, the pain commencing at a particular hour of the day, lasting a certain period and then disappearing completely for a time.

The pathology of neuralgia has not yet been clearly made out, and the causation of the disease is often extremely obscure. When we consider that the fifth nerve which supplies the teeth is distributed also to nearly the whole of the head and face, it is at least not difficult to understand that diseases of the teeth may act as frequent exciting causes of facial neuralgia. Various diseases of the teeth are capable of giving rise to neuralgic pain, but among all chronic inflammation of the pulp is the most frequent cause. In every case of facial neuralgia a careful examination of the teeth should be made.

It is not sufficient to take the patient's assurance that his teeth are not decayed, or that he does not suffer from the toothache. Patients are often unconscious of the presence of disease, and teeth which do not ache are frequently the excitants of neuralgic pain. Neither is it sufficient to cast a glance into the mouth for carious cavities. Each tooth should be separately explored with the aid of a probe and mouth mirror. Cavities hidden in the interstices or below the gum must be sought for, and the signs of necrosis, exostosis, inflammation, and thickening of the dental periosteum must not be overlooked.

Decayed and broken down wisdom teeth, common causes of neuralgia and especially of pain in the ear, are often, owing to their position, difficult to discover. They are in instances placed at the extremity of the alveolar ridge, and in the upper jaw are invisible except with a mouth mirror. In the lower jaw the wisdom teeth are commonly hidden by folds of the cheek or by overhanging gum. Decaying in many instances before they are completely erupted, these teeth after the destruction of their crowns by caries are occasionally quite invisible, and their presence in the sockets can be ascertained only by passing a probe through the small fistulous tract in the gum which covers them. A condition similar to this may also exist in the case of any other tooth; and buried roots, especially when the seat of exostosis, are the excitants of neuralgia in numerous instances. In cases of obstinate neuralgia it is proper to remove such diseased teeth as cannot be brought by treatment into a healthy state. It must be born in mind that all decayed teeth need not be condemned to extraction.

Neuralgia may be often guarded against by filling decayed cavities and protecting the sensitive structures of the teeth from irritation. In these operations care should be taken to avoid increasing the susceptibility of the teeth to change of temperature. For this purpose the oxychloride of zinc answers well, and in cases in which the pulp is exposed or protected only by a thin layer of dentine, the oxysulphate will answer better.

There are other surgical diseases besides those of the teeth which involve branches of the fifth nerve, and which may therefore originate neuralgia. The nerve, or its branches, may be compressed by tumors or may be affected by inflammation, exostosis, or necrosis of the bony canals through which they pass. Inflammation of the mucous membrane of the antrum may include the superior dental nerves. A foreign body may be imbedded in a branch of the nerve. The discovery and removal of the exciting cause must be the first care in dealing with neuralgia, but the treatment of the predisposing causes must not be overlooked.

By improving the general health the pain frequently disappears, although the exciting cause may remain. If the disease has been developed by any form of debility, or by malaria, full doses of quinine gives almost always good results, and where this drug fails, Fowlers' solution of arsenic will be good. In those cases in which the cause of neuralgia can not be discovered, an attempt can be made to destroy the excitability of the painful nerves. For this purpose electricity in various forms is employed. Cold which diminishes for a time the excitability of the nerves, may be applied to the skin by means of ice or evaporating lotions; and ointments of aconite or veratria, or lotions of belladona and chloroform, produce similar effects.

